# Chronic pain and foreign body sensation based on mesh placement in primary ventral hernia repair: a systematic review highlighting the evidence gap and a call to action

**DOI:** 10.1007/s00423-025-03671-2

**Published:** 2025-04-21

**Authors:** Usamah Ahmed, Jacob Rosenberg, Jason Joe Baker

**Affiliations:** Center for Perioperative Optimization, Department of Surgery, Copenhagen University Hospital - Herlev and Gentofte, Borgmester Ib Juuls Vej 1, Herlev, DK-2730 Denmark

**Keywords:** Ventral hernia, Onlay, Retromuscular, Preperitoneal, Intraperitoneal, Chronic pain

## Abstract

**Purpose:**

This systematic review aimed to investigate differences in chronic pain and foreign body sensation based on mesh placement, with recurrence as a secondary outcome.

**Methods:**

The review was registered in PROSPERO (ID: CRD42024592114), and searches were conducted in MEDLINE (PubMed), Embase Ovid, and Cochrane CENTRAL on October 3rd, 2024. Studies were included if they compared mesh placements, categorized as onlay, retromuscular, preperitoneal, or intraperitoneal onlay mesh (IPOM), in primary ventral hernia repairs in adults. Chronic pain (≥ 6 months post-surgery) and foreign body sensation were the primary outcomes. Randomized controlled trials (RCTs) and cohort studies were included, while incisional hernias alone and animal studies were excluded. Risk of bias was assessed using the Newcastle-Ottawa Scale for observational studies and Cochrane Risk of Bias 2 (RoB2) tool for RCTs. Due to significant heterogeneity, a meta-analysis was not feasible, and a narrative synthesis was provided.

**Results:**

A total of 6,562 records were screened, of which nine cohort studies and one RCT were included. Studies were heterogeneous and many did not aim to assess chronic pain as the primary outcome. Two studies reported significantly lower chronic pain rates with retromuscular mesh placement, but data pooling was not possible. Foreign body sensation could not be assessed because the only study reporting on this did not have extractable data.

**Conclusion:**

Currently, there is insufficient evidence to favor one mesh placement over another for chronic pain or foreign body sensation. While crude rates suggest that retromuscular and preperitoneal placements may result in less chronic pain than onlay and IPOM, the evidence remains very uncertain due to significant clinical and methodological heterogeneity. Further research is warranted.

## Introduction

Mesh placement is recommended for ventral hernia repair and has become the standard practice [1, 2]. Ventral hernia repair is among the most frequently performed elective abdominal surgeries, with various mesh placements, including onlay, preperitoneal, retromuscular, and intraperitoneal onlay mesh (IPOM) [3–5]. Historically, laparoscopic IPOM was the preferred minimally invasive approach, but its popularity has declined due to associations with rare but severe complications such as bowel adhesions and obstruction [6]. Different mesh placements require access to and positioning in different anatomical planes, each involving potential complications due to neurovascular injury, fascial dissection, or mesh-related issues [7]. Common patient concerns include chronic pain, foreign body sensation, and recurrence [8, 9]. Therefore, we need to clarify the best approach for mesh placement concerning chronic pain and foreign body sensation based on current published evidence. Moreover, this needs to be clarified for primary and incisional hernias separately as they are different hernia types with different risks of outcomes [10–12]. The 2020 guidelines from the American Hernia Society (AHS) and European Hernia Society (EHS) suggest preperitoneal or retromuscular mesh placement to reduce surgical site infection and recurrence rates [1]. However, these recommendations are based on weak evidence and low-quality evidence [1].

This systematic review aimed to investigate whether chronic pain and foreign body sensation differ depending on the mesh placement in adult patients undergoing primary ventral hernia repair. A secondary objective was to assess recurrence rates based on the mesh placement technique.

## Methods

### Study design

This systematic review was reported according to the Preferred Reporting Items for Systematic Reviews and Meta-Analyses (PRISMA 2020) guidelines, incorporating the Synthesis Without Meta-Analysis (SWiM) extension [13, 14]. The protocol for this review was pre-registered in the PROSPERO database under the registration ID CRD42024592114.

### Eligibility criteria

The eligibility criteria in this systematic review were adults (≥ 18 years old) who had undergone a primary ventral hernia repair with mesh. Regardless of the studies’ definitions, mesh placements were defined as onlay if positioned superficially to the anterior rectus fascia, retromuscular if placed between the rectus muscle and the posterior rectus fascia, preperitoneal if placed between the posterior rectus fascia and parietal peritoneum, and intraperitoneal if placed on the visceral side of the parietal peritoneum. Studies were required to include at least two groups with different mesh placements, with a minimum of 20 patients in each group. Studies that included both incisional and primary ventral hernia repairs were eligible only if the number of primary ventral hernia repairs exceeded the number of incisional hernia repairs. The review included randomized controlled trials (RCTs) and cohort studies. We excluded animal studies and studies with only parastomal, Spiegel, lumbar, incisional, complex, or giant hernias as well as studies only including component separation or using absorbable or biological mesh. Case series, case reports, reviews, and conference abstracts were also excluded.

The primary outcomes were chronic pain and foreign body sensation, with a minimum follow-up period of six months. Both outcomes were primarily assessed as binary outcomes (yes or no). The secondary outcome was recurrence, as defined by the authors of each included study.

### Information sources

The search was conducted on October 3rd, 2024, in MEDLINE (PubMed) from 1966, Embase Ovid from 1974, and Cochrane CENTRAL. Forward and backward citation searches were conducted on all included studies and relevant systematic reviews [15].

The search string was developed in collaboration with an information specialist. The search string for MEDLINE (PubMed) was: (((“ventral” OR epigastric* OR “umbilical” OR “abdominal”) AND hernia) OR “Hernia, Ventral“[Mesh]) AND (onlay OR premuscular OR sublay OR retromuscular OR retrorectus OR preperitoneal OR intraperitoneal OR IPOM OR underlay OR open OR robot* OR laparoscop* OR “Laparoscopes“[Mesh]) AND (pain OR foreign* OR “prom” OR “proms” OR “pro” OR “pros” OR “Patient-report*” OR “Patient report*” OR “self-report*” OR “self report*” OR “QOL” OR “Quality of Life” OR “Quality of Life“[Mesh] OR “Patient Reported Outcome Measures“[Mesh] OR “Self Report“[Mesh]). The search strategies used for Embase Ovid and Cochrane CENTRAL are detailed in the protocol uploaded to PROSPERO [16]. While the search was conducted in English, studies in other languages were not excluded. A re-run of the search was not performed prior to the final data synthesis.

### Selection process

Covidence [17] was used to first screen titles and abstracts and then full texts. Covidence automatically removed duplicates [17]. Two independent authors screened each title and abstract for eligibility. If discrepancies arose, the report was discussed till agreement, or a third author was included. If data on pain, foreign body sensation, and recurrence were described in the studies’ methods section, but not reported in the results, the contact author was contacted by email in an attempt to obtain missing data. To avoid duplicate populations, studies reporting data derived from the same database were included only once.

### Data collection process

Data were extracted from the reports by a single author in collaboration with another author. A pilot-tested data extraction sheet in Excel (Version 2308, Microsoft Office 365) was used for this process. The extracted data were validated twice by the same author to ensure accuracy. Any uncertainties that arose during data extraction were discussed and resolved within the author group.

### Data items

The data items extracted were the following: authors, year of publication, conflict of interest, study country, study design, study period, number of patients, percentage of females, age, hernia type, hernia defect width (if reported as area, this was converted to defect width from the formula $\:A=\pi\:\cdot{r}^{2}$), number of hernia types, surgical approach, mesh placement, use of the IPOM-plus technique [18], and method of fixation. No imputation was performed for missing data. If data were not reported numerically but presented graphically, they were extracted from the figures.

### Study risk of bias assessment

Bias assessment was conducted by two authors. Observational studies were evaluated using the Newcastle-Ottawa Scale, which assesses three domains: selection, comparability, and outcome [19]. Within the comparability domain, a star was awarded if the study included only primary ventral hernia repairs. A star could also be awarded if the study demonstrated comparability in other inclusion criteria, such as hernia defect size. Patient characteristics, such as age, sex, and smoking status, were likewise reviewed for apparent differences; if significant differences were observed, a second star was not awarded in this domain. For the outcome domain, chronic pain and foreign body sensation were self-reported measures, making it impossible to award a star. Consequently, the highest achievable score for a study was eight stars. RCTs were assessed with the Cochrane Risk of Bias 2 (RoB2) tool, with the following domains: bias due to the randomization process, deviations from intended interventions, missing outcome data, measurement of outcomes, and selection of reported results [20]. The RoB2 assessment was supplemented with a guidance document [21]. To assess reporting bias, a bubble plot was created, and discrepancies in the distribution of data points were qualitatively evaluated for potential asymmetry, which could indicate reporting bias.

### Effect measures

Outcomes were reported as crude rates from the total number of patients at the time of follow-up. Chronic pain was defined if the VAS score was ≥ 3. Chronic pain and foreign body sensation were also included when no intensity level was specified. Chronic pain reported during activity was prioritized, as it was assumed that patients experiencing pain at rest would also report pain during activity. If a study only reported pooled chronic pain intensity, this was included as well. Recurrence was considered at any time point reported within the follow-up period for the included studies. This inclusive approach was selected to accommodate variability in follow-up durations across studies. Missing data were excluded from the denominator unless the included studies explicitly addressed their treatment. For studies that did not report *p-*values, these were calculated to assess statistical significance between rates of chronic pain associated with different mesh placements within each study with Pearson’s chi-square test or Fisher’s exact test.

### Synthesis methods

A meta-analysis, as originally planned in the protocol, was deemed unfeasible due to high clinical and methodological heterogeneity across the included studies. For instance, not all studies directly compared different mesh placements; instead, this information had to be extracted from the data. Additionally, chronic pain was not consistently reported as the primary outcome, and some studies employed a mix of IPOM and IPOM-plus techniques. Furthermore, mesh placements (retromuscular, preperitoneal, and IPOM) were performed using both open and laparoscopic techniques, and patient characteristics, such as the percentage of females, varied widely (e.g., 9–67%). Given these factors, a narrative synthesis was conducted instead, reporting crude rates to accommodate the significant heterogeneity. Studies were tabulated and ranked by the year of publication.

### Certainty assessment

The GRADE framework was used to assess the certainty of evidence for each outcome considering the risk of bias, consistency, indirectness, imprecision, and publication bias. Certainty ratings were assigned as high, moderate, low, or very low based on predefined criteria [22].

## Results

### Study selection

**Fig. 1:**
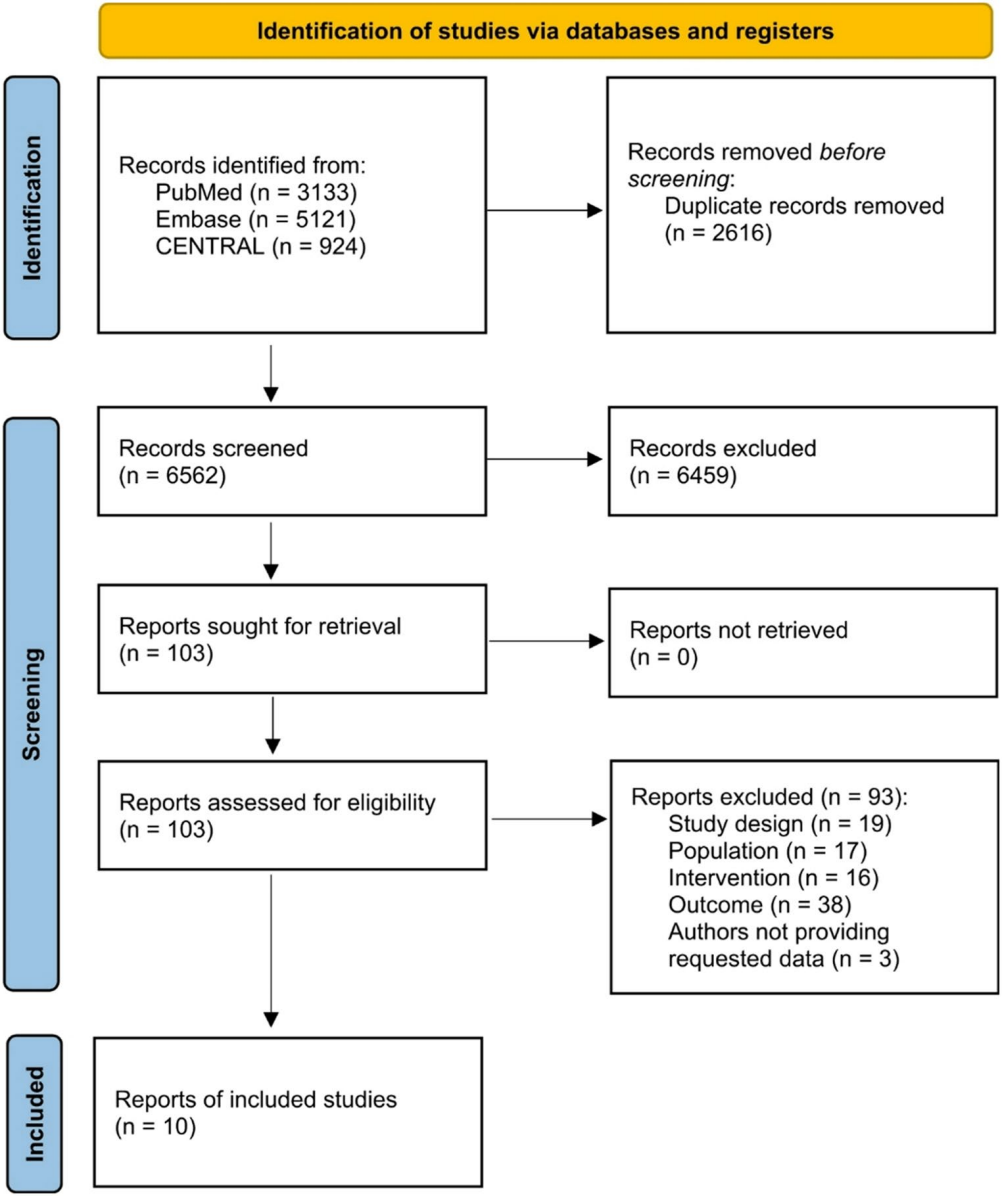
PRISMA flowchart. n: number of reports

A total of 6,562 records were screened by title and abstract (Fig. 1). Ultimately, ten studies were included in this systematic review, reporting on 5,968 patients [23–32]. Of these, eight studies contributed data for crude rate calculations, encompassing 5,790 patients [25–32], while two additional studies reported on pooled pain intensity in 178 patients [23, 24].

Initially, 13 reports appeared to meet the eligibility criteria, but only ten were included as explained in the following. Of the 13 reports, ten mentioned in their methods section that data relevant to our review had been recorded, but these data were not reported in the published paper. Nine study authors were contacted of which three reports had failed to report all outcomes of interest [28–30], three pooled mesh placement data [25, 33, 34], and two did not report crude rates as binary outcome [23, 24]. One report included both incisional and primary hernias but did not specify their proportions [35]. One study author was not contacted due to the study being more than ten years old [32]. Among the authors contacted, two responded. One provided the requested data [25], while the other reported that the data had been deleted [33]. Seven authors did not respond [23, 24, 28–30, 34, 35]. As a result, two reports were excluded due to pooling of variables [33, 34], and one was excluded due to pooling incisional and primary hernias without specifying the ratio [35]. No additional studies were identified through forward or backward citation searching.

### Study characteristics

Nine cohort studies [23–25, 27–32] and one RCT [26] were included (Table 1). All cohort studies collected follow-up data prospectively. Seven studies exclusively focused on primary ventral hernias [23–25, 28–30, 32], while the remaining three included both primary and incisional hernias [26, 27, 31]. Most studies reported a mean hernia size of 2–4 cm, although one had defects up to 10 cm in width [31], and another included hernia sizes up to 8 cm, although the median defect size was 1 cm [32]. One study categorized hernias > 4 or ≤ 4 cm but did not provide further details, with most hernia sizes being ≤ 4 cm [25]. The age ranged from 40 to 50 years, but two studies did not report participant ages [28, 29]. The male-to-female ratio varied; most studies reported an approximately equal distribution, but one study included only 9% females [23], while another included 67% females [32]. Fixation methods also varied, and six studies reported fixation methods, which ranged from sutures [31] to tacks combined with sutures [23–26, 30]. Tacks and sutures also varied, whether absorbable or permanent. Surgical approaches also varied: six studies compared open versus laparoscopic techniques for onlay, preperitoneal, retromuscular, and IPOM placements [23, 25, 27–30]. One RCT compared laparoscopic IPOM with endoscopic eTEP (retromuscular) mesh placement [26]. Two studies investigated open techniques only with onlay, preperitoneal, and IPOM placements [31, 32]. Another study compared laparoscopic IPOM with robotic TAPP (preperitoneal) mesh placement [24]. Three studies explicitly used the IPOM-plus technique [23, 24, 30], and three studies did not use IPOM-plus [26, 27, 31]. One study used a mix of IPOM-plus and IPOM [25], and three studies did not specify whether IPOM-plus or standard IPOM was used [28, 29, 32].

**Table 1:** Study characteristics

Author and Year	Study design	*N* patients	Female, %	Hernia type	Defect width
Ertekin 2024 [23]	Cohort	67	9	Umbilical	4.1 cm ^a^
Bindal 2024 [24]	Cohort	111	57	Primary	2.0 cm ^a^
Schjøth-Iversen 2023 [25]	Cohort	300	38	Primary	≤ 4 cm ^c^
Jain 2022 [26]	RCT	60	57	Primary (31) and incisional (29)	3.7 cm ^a^
Kalyan 2022 [27]	Cohort	50	46	Paraumbilical (17), umbilical (11), epigastric (11), and incisional (11)	3.0 cm ^a^
Köckerling 2021 [28]	Cohort	2,702	48	Epigastric	NR
Köckerling 2021 [29]	Cohort	2,310	NR	Umbilical	< 2 cm ^c^
van den Dop 2020 [30]	Cohort	99	58	Epigastric	2.2 cm ^a^
Shah 2019 [31]	Cohort	180	46	Paraumbilical (23), umbilical (63), epigastric (9), and incisional (85)	≤ 10 cm ^c^
Erritzøe-Jervild 2013 [32]	Cohort	89	67	Umbilical (NR) and epigastric (NR)	1 cm ^b^

Numbers in parentheses indicate the number of each hernia type. N: number. RCT: randomized controlled trial. NR: not reported. a: mean. b: median. c: size reported without mean or median

### Risk of bias

For chronic pain, observational studies had a median bias score of six (range 4–7) out of eight possible stars on the Newcastle-Ottawa Scale (Table 2). Only one study also assessed pre-operative pain, earning four stars in the selection domain [23]. In the comparability domain, two studies earned two stars for only including specific hernia types and sizes [24, 29], five received one star [23, 25, 28, 30, 32], and two received none due to pooling incisional and primary hernias [27, 31]. No stars were awarded for the assessment of the outcome domain of the Newcastle-Ottawa Scale, as chronic pain was self-reported. One study did not report the time chronic pain was assessed and did not get a star [31]. All other studies reported chronic pain six months or later. Eight studies had adequate loss to follow-up resulting in a star. One study excluded non-responders, which was noted as a limitation [23].

The one study reporting on foreign body sensation [30] received only five stars in total because of failure to assess pre-operative presence of foreign body sensation and significant loss to follow-up (46%).

**Table 2:** Newcastle-Ottawa scale (NOS) evaluation

Study	Selection				Comparability	Outcome			Total score (max. 8)
	1	2	3	4	5	6	7	8	
Ertekin 2024 [23]	★	★	★	★	★	NA	★	--	6
Bindal 2024 [24]	★	★	★	--	★★	NA	★	★	7
Schjøth-Iversen 2023 [25]	★	★	★	--	★	NA	★	★	6
Kalyan 2022 [27]	★	★	★	--	--	NA	★	★	5
Köckerling 2021 [28]	★	★	★	--	★	NA	★	★	6
Köckerling 2021 [29]	★	★	★	--	★★	NA	★	★	7
van den Dop 2020 [30]	★	★	★	--	★	NA	★	CP = ★ FBS = --	CP = 6 FBS = 5
Shah 2019 [31]	★	★	★	--	--	NA	--	★	4
Erritzøe-Jervild 2013 [32]	★	★	★	--	★	NA	★	★	6

NA: not applicable. CP: chronic pain. FBS: foreign body sensation. (1): Representativeness of the exposed cohort. (2): Selection of the non-exposed cohort. (3): Ascertainment of exposure. (4): Demonstration that outcome of interest was not present at start of study. (5): Comparability of cohorts on the basis of the design or analysis. (6): Assessment of outcome. (7): Was follow-up long enough for outcomes to occur. (8): Adequacy of follow up of cohorts

**Fig. 2:**
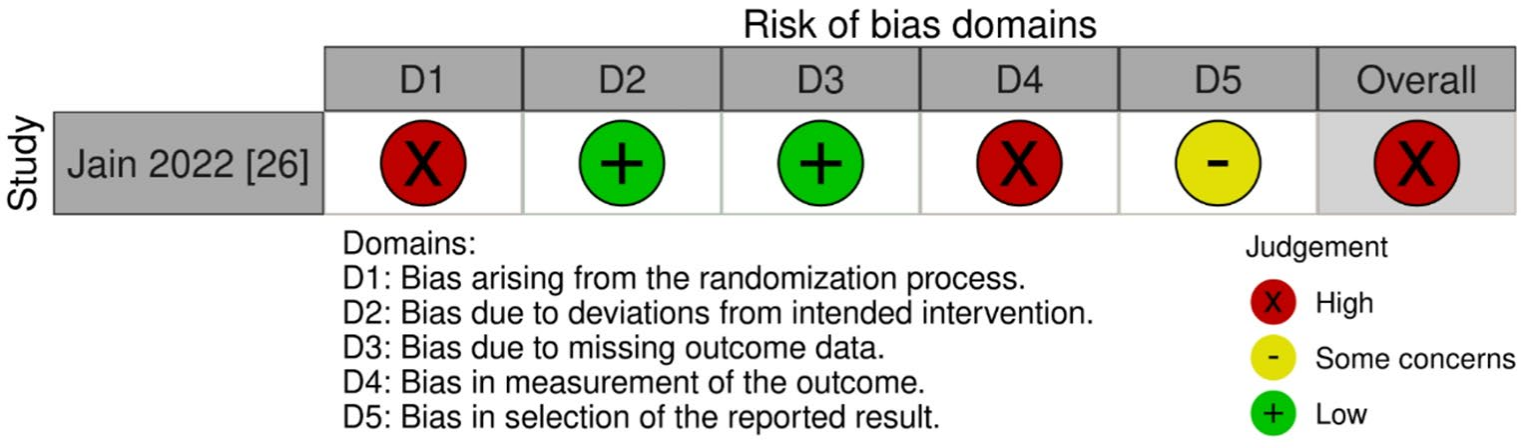
Cochrane Risk of Bias 2 (RoB2) tool assessment of randomized controlled trials [45]

The one RCT that was included was judged as having a generally high risk of bias (Fig. 2) [26]. Block randomization with equally sized blocks (*n* = 8) allowed allocation prediction, raising concerns about the randomization process [21]. Lack of information on participant blinding also introduced bias, as participants were the assessors of chronic pain, and assessment of the outcome could have been influenced by knowledge of intervention received. These factors contributed to a high overall risk of bias.

### Chronic pain

The results from the included studies are summarized in Table 3. The reported crude rates of chronic pain varied by mesh placement: onlay 8–24%, retromuscular 3–13%, preperitoneal 4–6%, and IPOM 0–20%. Only two studies reported significant differences between mesh placements: one found lower chronic pain rates with retromuscular placement compared with IPOM (*p* = 0.0478) [28]. Another study found lower chronic pain rates for retromuscular placement versus onlay (*p* = 0.0069) but did not define when pain was considered chronic, and only crude rates were provided [31].

**Table 3:** Crude rates for chronic pain

Author	Follow up, years	Chronic pain, *n*/*N* (%)				
		Onlay	Retromuscular	Preperitoneal	IPOM	
Schjøth-Iversen 2023 [25]	0.5^c^	1/8 (13)	--	4/62 (6)	20/230 (9)	M
Jain 2022 [26]	0.5^c^	--	4/30 (13)	--	6/30 (20)	
Kalyan 2022 [27]	0.5^c^	2/25 (8)	--	--	0/25 (0)	
Köckerling 2021 [28]	1^c^	17/147 (12)	96/1079 (9)	--	171/1476 (12)	?
Köckerling 2021 [29]	1^c^	23/249 (9)	--	--	162/2061 (8)	?
van den Dop 2020 [30]	6.3^b^	--	--	2/57 (4)	1/42 (2)	+
Shah 2019 [31]	NR	15/90 (17)	3/90 (3)	--	--	
Erritzøe-Jervild 2013 [32]	3^b^	5/21 (24)	--	--	7/68 (10)	?

n/N: number of patients with chronic pain out of all participants followed up. NR: follow up not reported but assumed to be 1 year as recurrence is assessed at 1 year in the same study. IPOM: intraperitoneal onlay mesh. +: IPOM plus.?: not reported if IPOM plus. M: Mix of IPOM and IPOM plus. b: median. c: reported without mean or median

**Fig. 3:**
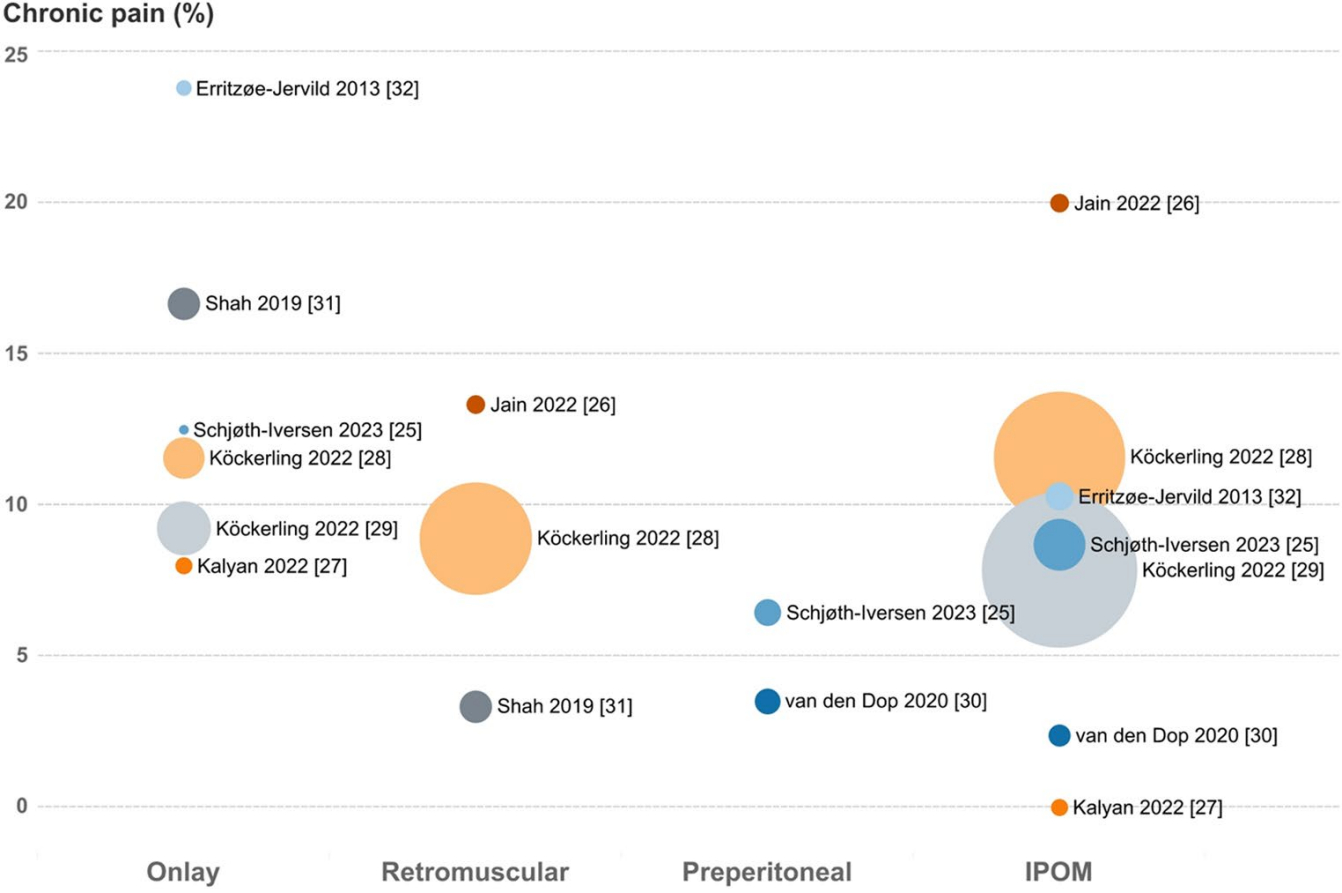
Bubble chart. Size of each bubble represents the number of patients followed up for each mesh placement in each study. Center of the bubble indicates the crude rate of chronic pain (%) on the y-axis. Each bubble color represents the same study. Corresponding study and reference are placed to the right of the bubble

Measurement of chronic pain varied across studies. Most reported it as binary (yes/no), while two used visual analogue scale (VAS) [24, 25] and one used SF-36 [23]. Among studies reporting pooled pain intensity, one found that chronic pain, measured by VAS, was significantly lower in preperitoneal placement compared to IPOM placement after one year [24]. Follow-up times and assessment methods also varied, including questionnaires and clinical follow-ups. Figure 3 illustrates the relationship between study size and results, with smaller studies showing more extreme chronic pain rates and larger studies reporting more moderate rates. Not all included studies evaluated chronic pain as a primary outcome [27–29, 31]. Some reported it as part of comparisons between open and laparoscopic techniques [23, 27, 30], while others reported crude rates without adjustments [28, 29]. One author provided unadjusted rates after being contacted by email [25].

Crude rates suggested that retromuscular and preperitoneal mesh placements may have little to no effect on reducing the risk of chronic pain, but the evidence is very uncertain. According to the GRADE assessment, the starting point for evidence is low certainty due to the predominance of nine observational studies, as all are assessed for bias with Newcastle-Ottawa Scale. The included RCT was initially rated as high-certainty evidence because of its design as an RCT, but it was subsequently downgraded due to high risk of bias under the RoB2 assessment. Further downgrades were made for heterogeneity, indirectness (i.e., inclusion of incisional hernias and lack of focus on chronic pain as a primary outcome), and imprecision (with crude rates supporting both benefit and harm), resulting in very low certainty of evidence.

### Foreign body sensation

One study measured foreign body sensation stratified by mesh placements [30]. This was not reported as a binary outcome but as part of the Carolina Comfort Scale [36]. No significant differences were observed in the Carolina Comfort Scale scores between preperitoneal and IPOM placements after at least one year. The certainty of evidence is very low, primarily due to reliance on a single study and the use of a composite scale rather than a binary outcome measure.

### Recurrence

Recurrence was reported by all included studies over the entire follow-up period, with a median follow-up of one year (range 0.5 to 5 years). Recurrence rates varied by mesh placement: onlay 0–10%, retromuscular 0–2%, preperitoneal 0–7%, and IPOM 0–10% (Table 4). Only one study found a significant difference between mesh placements, reporting fewer recurrences with retromuscular and IPOM placements compared to onlay (*p* < 0.001) [28]. No significant difference was observed between retromuscular and IPOM placements (*p* = 0.058) [28]. Some studies did not specify how recurrence was diagnosed, such as whether it was identified during clinical follow-ups or self-reported in questionnaires [24, 27, 31]. One study used a Kaplan-Meier plot to display recurrence rates, and these were calculated from the percentages and total numbers in the onlay and IPOM groups, respectively [32].

**Table 4:** Crude rates for recurrence

Author	Follow up, years	Recurrence, *n*/*N* (%)					
		Onlay	Retromuscular		Preperitoneal	IPOM	
Ertekin 2024 [23]	1^c^	0/32 (0)		--	--	2/35 (6)	+
Bindal 2024 [24]	1^c^	--		--	0/33 (0)	0/78 (0)	+
Schjøth-Iversen 2023 [25]	5^b^	0/8 (0)		--	3/62 (5)	20/230 (9)	M
Jain 2022 [26]	0.8^a^	--		0/30 (0)	--	0/30 (0)	
Kalyan 2022 [27]	0.5^c^	1/25 (4)		--	--	0/25 (0)	
Köckerling 2021 [28]	1^c^	14/147 (10)		18/1079 (2)	--	42/1476 (3)	?
Köckerling 2021 [29]	1^c^	2/249 (< 1)		--	--	29/2061 (1)	?
van den Dop 2020 [30]	0.6^a^	--		--	4/57 (7)	1/42 (2)	+
Shah 2019 [31]	1^c^	3/90 (3)		0/90 (0)	--	--	
Erritzøe-Jervild 2013 [32]	3^b^	2/21 (8)		--	--	7/68 (10)	?

n/N: number of recurrences out of all participants followed up. IPOM: intraperitoneal onlay mesh. +: IPOM plus.?: not reported if IPOM plus or not. M: Mix of IPOM and IPOM plus. a: mean. b: median. c: reported without mean or median

## Discussion

Based on unadjusted crude rates, retromuscular and preperitoneal mesh placements may have little to no effect on reducing the risk of chronic pain, given the very low certainty of evidence. Foreign body sensation could not be assessed, as it was included in only one study and reported as part of the Carolina Comfort Scale rather than as a binary outcome. Retromuscular and preperitoneal mesh placements seem to have the lowest rates of recurrence based on crude rates. However, due to the heterogeneity of the included studies and the inability to pool data, no definitive conclusion can be drawn about the superiority of any specific mesh placement.

To our knowledge, no other systematic review has specifically focused on the association between mesh placement in primary ventral hernia repair and chronic pain or foreign body sensation. However, a systematic review from 2018 evaluating mesh repair versus non-mesh sutured repair, with recurrence as the primary outcome, highlighted a similar challenge, noting insufficient data to determine optimal mesh placement in open umbilical hernia repair [37]. A systematic review from 2013 found that retromuscular and IPOM placements tended to lower overall complication and recurrence rates compared to onlay and inlay placements [38]. However, this review pooled data from both incisional and primary ventral hernias and included both synthetic and biological meshes, limiting its applicability. An updated version from 2018 reported no major new findings [39]. Another systematic review with meta-analysis from 2017 suggested that sublay (retromuscular and preperitoneal) placements were superior in terms of complications and showed a tendency toward lower recurrence and surgical site infection rates compared to onlay and IPOM placements [4]. However, the odds ratio for recurrence was inconsistent with its confidence interval, making it unclear if the findings were statistically significant. Furthermore, the review pooled primary and incisional ventral hernias and included studies that had several limitations, including unclear patient allocation, insufficient detail on randomization strategies, and a lack of blinding. These factors further complicate the interpretation of findings and the determination of optimal mesh placement.

### Strengths and limitations

The strength of this systematic review lies in its comprehensive search strategy, developed with the assistance of an information specialist, which included broad search strings and no language restrictions. The findings were summarized and assessed for quality using the GRADE framework. This review focused on chronic pain and foreign body sensation, both of which are important concerns for patients undergoing hernia repair [8, 9]. By identifying high levels of heterogeneity and gaps in evidence, this review emphasizes the need for standardized, high-quality studies. The decision to refrain from undertaking a meta-analysis due to high heterogeneity prevented oversimplification of data, reducing the risk of misrepresenting the relationship between mesh placement and chronic pain and foreign body sensation. However, there were also several limitations in this study. Two of the included studies reported significantly lower chronic pain rates for retromuscular mesh placement compared to IPOM and onlay [28, 31], but these findings were based on unadjusted crude rates, leaving potential confounders unaddressed, thus limiting the robustness of the findings. One of these studies included epigastric hernias but did not report the mean defect width, mean age, or detailed outcomes beyond trends in surgical techniques for epigastric hernia repair over time [28]. Many studies compared open, laparoscopic, and robotic approaches with different mesh placements, making it unclear whether the surgical approach or the mesh placement was the primary factor influencing chronic pain rates. Variations in fixation methods and the use of IPOM plus may also affect outcomes [40, 41]. Additionally, the type and weight of mesh, which might also affect chronic pain and foreign body sensation, were not consistently reported [42]. Finally, one study did not specify when chronic pain was assessed [31]. While recurrence was evaluated at one year post-surgery, it is unclear if chronic pain was assessed at the same time. Despite this uncertainty, the study was included in the review under the assumption that the follow-up timelines were aligned.

### Perspectives

Patient-reported outcomes, such as chronic pain and foreign body sensation, are important for evaluating the true success of hernia repair. Current guidelines suggest preperitoneal or retromuscular mesh placement for primary ventral hernia repair [1]. However, five years after their publication, this review finds that current evidence on chronic pain and foreign body sensation remains insufficient, highlighting the need for improved reporting and analysis. The lack of evidence on foreign body sensation as a standalone measure significantly limits the ability to address patients’ concerns. Future studies should prioritize reporting foreign body sensation as a separate outcome to fill this gap. While retromuscular and preperitoneal mesh placements may show promise in reducing chronic pain, the certainty of evidence is very low, underscoring the need for well-designed comparative studies to evaluate these long-term outcomes. Ideally, large-scale, multicenter RCTs with long-term follow-up periods are needed to generate high-quality evidence. Additionally, robust observational studies employing matching, real-world data analysis, and long-term registries may serve as a suitable alternative [43]. Encouragingly, research aimed at better understanding patient-reported outcomes in hernia repair appears to be underway [44]. Such efforts will be critical in advancing the evidence base for hernia repair, guiding clinical decision-making, and ultimately enhancing patient care.

## Conclusion

Currently, there is insufficient evidence to favor one mesh placement over another regarding chronic pain and foreign body sensation. While crude rates suggest that retromuscular and preperitoneal placements may result in less chronic pain than onlay and intraperitoneal placements, we lack confidence in the current evidence due to significant clinical and methodological heterogeneity. Additionally, the lack of standalone data prevents any conclusions on foreign body sensation. This calls for well-designed comparative studies on mesh placement with patient-reported outcomes to improve patient care in primary ventral hernia repair.

## Data Availability

The data that support the findings of this study are available from the corresponding author, UA, upon reasonable request.
